# Experiences and lessons learned from the real-world implementation of an HIV recent infection testing algorithm in three routine service-delivery settings in Kenya and Zimbabwe

**DOI:** 10.1186/s12913-021-06619-6

**Published:** 2021-06-22

**Authors:** Mariken M. de Wit, Brian Rice, Kathryn Risher, Susie Welty, Wanjiru Waruiru, Sitholubuhle Magutshwa, John Motoku, Daniel Kwaro, Benard Ochieng, Georges Reniers, Frances Cowan, George Rutherford, James R. Hargreaves, Gary Murphy

**Affiliations:** 1 London School of Hygiene and Tropical Medicine, London, UK; 2 University of California, San Francisco, USA; 3 Centre for Sexual Health and HIV/AIDS Research, Harare, Zimbabwe; 4 Eastern Deanery AIDS Relief Programme, Nairobi, Kenya; 5 Kenya Medical Research Institute, Kisumu, Kenya; 6 Liverpool School of Tropical Medicine, Liverpool, UK; 7 Independent consultant in HIV laboratory diagnostics, London, UK

**Keywords:** HIV, Recency testing, RITA, Zimbabwe, Kenya, Implementation

## Abstract

**Introduction:**

Testing for recent HIV infection can distinguish recently acquired infection from long-standing infections. Given current interest in the implementation of recent infection testing algorithms (RITA), we report our experiences in implementing a RITA in three pilot studies and highlight important issues to consider when conducting recency testing in routine settings.

**Methods:**

We applied a RITA, incorporating a limited antigen (LAg) avidity assay, in different routine HIV service-delivery settings in 2018: antenatal care clinics in Siaya County, Kenya, HIV testing and counselling facilities in Nairobi, Kenya, and female sex workers clinics in Zimbabwe. Discussions were conducted with study coordinators, laboratory leads, and facility-based stakeholders to evaluate experiences and lessons learned in relation to implementing recency testing.

**Results:**

In Siaya County 10/426 (2.3%) of women testing HIV positive were classified as recent, compared to 46/530 (8.7%) of women and men in Nairobi and 33/313 (10.5%) of female sex workers in Zimbabwe. Across the study setting, we observed differences in acceptance, transport and storage of dried blood spot (DBS) or venous blood samples. For example, the acceptance rate when testing venous blood was 11% lower than when using DBS. Integrating our study into existing services ensured a quick start of the study and kept the amount of additional resources required low. From a laboratory perspective, the LAg avidity assay was initially difficult to operationalise, but developing a network of laboratories and experts to work together helped to improve this. A challenge that was not overcome was the returning of RITA test results to clients. This was due to delays in laboratory testing, the need for multiple test results to satisfy the RITA, difficulties in aligning clinic visits, and participants opting not to return for test results.

**Conclusion:**

We completed three pilot studies using HIV recency testing based on a RITA in Kenya and Zimbabwe. The main lessons we learned were related to sample collection and handling, LAg avidity assay performance, integration into existing services and returning of test results to participants. Our real-world experience could provide helpful guidance to people currently working on the implementation of HIV recency testing in sub-Saharan Africa.

## Introduction

To identify populations and areas where HIV transmission is occurring, it is helpful to distinguish among newly diagnosed persons those who have acquired infection recently from those with a longer-standing infection. Several HIV recency tests have been developed to make such a distinction, including the HIV limiting antigen (LAg) avidity assay [1, 2]. Using LAg avidity assay test results alone (i.e. in the absence of viral load and antiretroviral therapy (ART) testing) to determine recency status is associated with an unacceptably high proportion false recent results (also known as false recency rate), which can lead to overestimation of HIV incidence without appropriate adjustment [3]. Recent infection testing algorithms (RITA) combine multiple test results to improve the classification of recent infections and decrease the proportion false recent. Since 2015, the United Nations Joint Programme on HIV/AIDS (UNAIDS) and the World Health Organization (WHO) have recommended the inclusion of viral load testing in RITAs to estimate incidence [4]. A study conducted in Kenya and South Africa showed that inclusion of ART exposure status in the RITA may further improve incidence estimates [5]. While these additional steps improve the accuracy of incidence estimates, they also make implementation of the RITA more challenging.

RITAs have been used in several settings, for example in trials [6], routine surveillance [7, 8] and in population surveys in sub-Saharan Africa [9–12]. In 2019, the U.S. President’s Emergency Plan for AIDS Relief (PEPFAR) called for recency testing to be implemented among all newly diagnosed individuals across all sites in supported countries that had achieved certain basic progress goals [13]. In some countries, recency test results are returned to participants, based on either the LAg avidity assay or the full RITA [14]. Previous studies have outlined some of the issues that need to be considered when conducting recency testing and when interpreting test results. These issues include differences in recency classification by sample type [15], the effect of storage temperature on dried blood spot (DBS) samples [16], and the need to include multiple tests in a RITA [3]. However, to the best of our knowledge, there are no published studies about real-word experiences in implementing recency testing in routine service-delivery settings.

We conducted three independent, but linked, pilots of HIV recency testing in a variety of routine service-provision contexts in Kenya and Zimbabwe in 2018. The three study settings were antenatal clinics providing prevention of mother-to-child HIV transmission services in Siaya County, Kenya, in HIV testing and counselling clinics in Nairobi, Kenya, and in a national programme for female sex workers in Zimbabwe. Here, we discuss our experiences with the implementation of a RITA and share lessons learned to inform other initiatives aiming to integrate recency testing into their routine programme services.

## Methods

### Study population

The three pilot studies were conducted in different routine service-provision settings (Table 1). In Siaya County, Kenya we tested pregnant women aged 13 years or older who tested HIV positive at their antenatal care visit at one of 14 antenatal care facilities in Gem subcounty. To determine HIV recency status, the Maxim HIV-1 LAg-Avidity enzyme-linked immunoassay (EIA) for venous blood was used. Recency test results were not returned to the participants. This pilot was conducted in collaboration with the Kenya Medical Research Institute (KEMRI), and participants were linked to the KEMRI/Centers for Disease Control and Prevention (CDC) Siaya Health and Demographic Surveillance Site (Siaya HDSS).

**Table 1:** Three pilots of HIV recent infection testing in routine service settings (adapted from [17]

Siaya County, Kenya	Nairobi, Kenya	Sisters with a Voice, Zimbabwe
**Study population**	**Study population**	**Study population**
Pregnant women seeking antenatal care in 14 medical facilities	Clients attending any of the 14 EDARP HTC facilities	FSW attending one of six static facilities of the Sisters with a Voice Programme
**Collaborative partner**	**Collaborative partner**	**Collaborative partner**
Kenya Medical Research Institute (KEMRI) and the KEMRI/CDC Siaya HDSS	Eastern Deanery AIDS Relief Programme (EDARP)	Centre for Sexual Health and HIV AIDS Research Zimbabwe (CeSHHAR-Zimbabwe)
**Study period**	**Study period**	**Study period**
February – November 2018	March – November 2018	June – November 2018
**Assay**	**Assay**	**Assay**
Maxim HIV-1 LAg-Avidity EIA venous blood	Maxim HIV-1 LAg-Avidity EIA DBS	Maxim HIV-1 LAg-Avidity EIA venous blood & Maxim HIV-1 LAg-Avidity EIA DBS
**Inclusion criteria**	**Inclusion criteria**	**Inclusion criteria**
• Women aged 13 or older seeking antenatal care in one of the included medical facilities in Siaya County • Willing and able to provide informed consent • Received an HIV-antibody positive test result	• Aged 18 or older • Unknown HIV status prior to visit • Attending an EDARP HTC facility • Willing and able to provide informed consent • Received an HIV-antibody positive test result, or presumptive positive	• FSW aged 18 or older • Willing and able to provide informed consent • Received an HIV-antibody positive test result
	**Exclusion criteria**	**Exclusion criteria**
	• Indeterminate HIV result • Not willing to enrol on follow-up at facility • Taking pre-exposure prophylaxis	• Indeterminate HIV result • Prior history of testing HIV-positive (> 1 year ago) • On ART
**Specimen collection and testing**	**Specimen collection and testing**	**Specimen collection and testing**
• Facility nurse or laboratory phlebotomist drew a maximum of 10 ml of venous blood • Samples packed and transferred to KEMRI-Centre for Global Health Research HIV Research Laboratory in Kisumu on a daily basis where they were processed and stored for testing	Study nurse drew 6 mL of venous blood collected in an ethylenediaminetetraacetic (EDTA) tube and a pipette was used to dispense venous blood on two Whatman™ 903 Snap-Apart Cards with 5 dried blood spots (DBS) of 70 μL each, for a total of 10 filled spots per participant	• Study or clinic nurse drew venous blood collected in an ethylenediaminetetraacetic (EDTA) tube. • DBS samples were collected from fingerprick on one Whatman™ 903 Snap-Apart Cards with 5 dried blood spots (DBS) of 70 μL. • Both samples were packed and transferred to laboratory in Harare within 36 h and stored at -20C or below prior to testing

In Nairobi, Kenya, we tested clients who tested HIV positive at any of 14 Eastern Deanery AIDS Relief Programme (EDARP) HIV testing and counselling facilities. EDARP has been providing community-based HIV and tuberculosis prevention, testing, treatment and care services in eastern Nairobi for the past 24 years [18]. Testing was done using Maxim HIV-1 LAg-Avidity EIA assay on dried blood spots (DBS). Results were returned to the client at their next clinic visit. Detailed methods and results from this pilot have been described elsewhere [19].

In Zimbabwe, our study focused on female sex workers testing HIV positive at any of six static sites of the Sisters with a Voice programme. Both venous blood samples and capillary DBS samples were taken. When a woman preferred not to give a venous blood sample, only a DBS was taken. HIV recency status was determined using Maxim HIV-1 LAg-Avidity EIA assays for venous blood as well as for DBS as both samples were taken. We compared acceptance rates between DBS and venous blood using McNemar’s test. Results of venous blood tests were to be made available at Sisters clinics within 2 weeks for timely return to participants. This pilot was conducted in collaboration with the Centre for Sexual Health and HIV/AIDS Research (CeSHHAR). CeSHHAR, on behalf of the Zimbabwean National AIDS Council and Ministry of Health and Child Care, runs the national Sisters with a Voice programme for female sex workers, providing a range of HIV-related services [20]. The three studies were linked through shared protocol design, and through joint coordination via a cross-pilot working group. The working group held bi-weekly conference calls which provided a platform for shared learnings and raising and responding to questions and issues as they arose.

### Recent infection testing algorithm

**Fig. 1:**
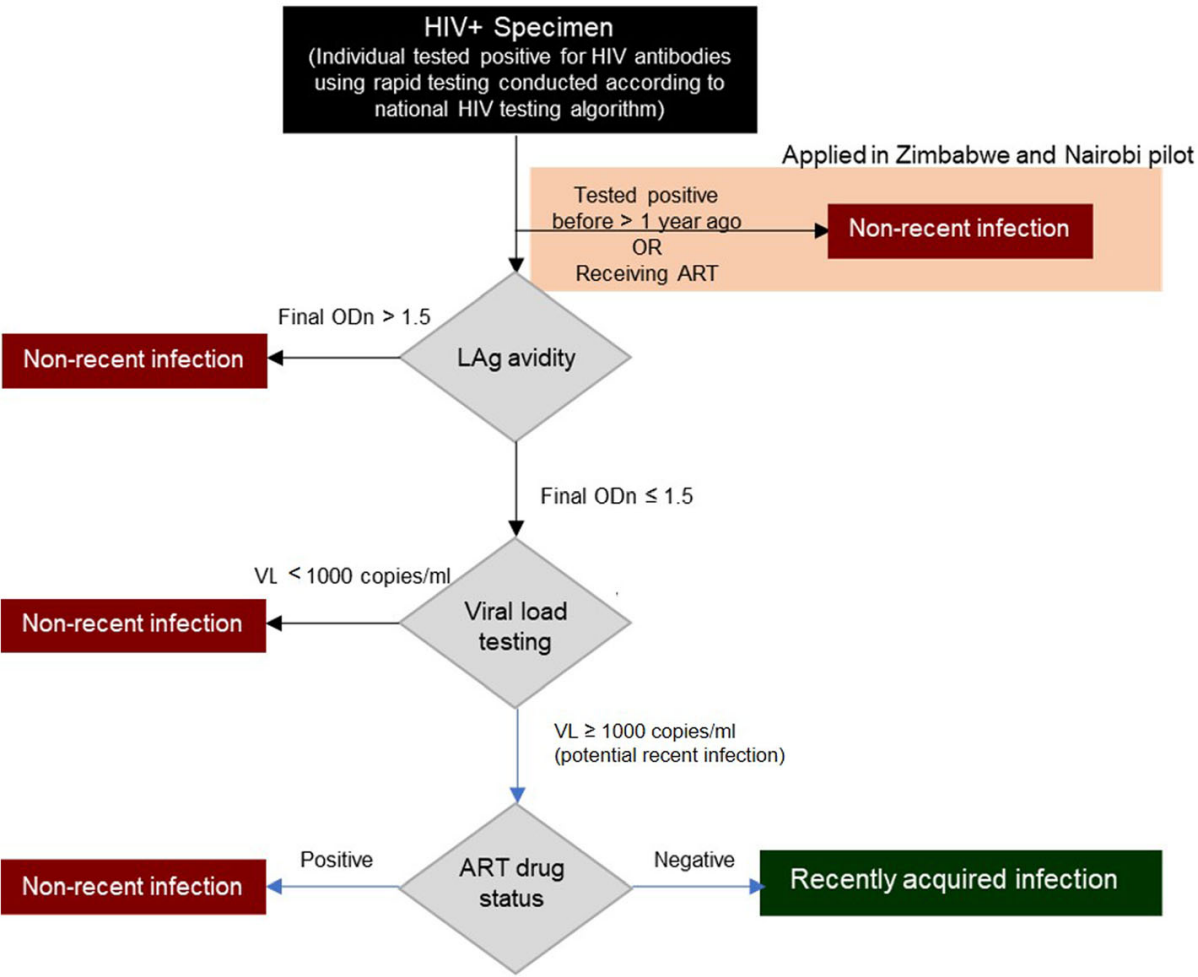
Recency testing algorithm (RITA) as applied in the three pilots (adapted from [17]

Figure 1 presents the RITA we integrated into routine service. All individuals who were tested HIV positive were assessed for eligibility. In Nairobi and Zimbabwe, we did not recruit individuals for assessment of recent infection if they self-reported a prior positive HIV antibody test (in Zimbabwe this had to be > 1 year ago) or if they reported taking ART. We classified these individuals as having a non-recent infection. We conducted LAg avidity testing on the remaining participants and classified samples with a normalized optical density (ODn) > 1.5 as long-standing infections. For samples with an ODn ≤1.5, we conducted viral load testing and classified those with a viral load < 1000 as long-standing infections and those with a viral load ≥ 1000 copies/mL as potentially positive for recent infection. We further characterised potentially positive samples by ART status determined by testing for the presence of ART metabolites (Nairobi), checking the clinic record (Siaya County), or self-report (Zimbabwe) and classified samples from people on ART as long-standing infections and those from people not on ART as positive for recent infection.

### LAg avidity assay

Recency assays are available for venous blood and DBS samples [21–24]. Sample collection via DBS is regarded as a simpler and more efficient method than venous blood sampling, because blood drops are easier to collect and DBS cards can be transported and stored dry at room temperature. Venous blood results are seen as more reliable (as they are less sensitive to haematocrit levels than DBS) but require venepuncture, processing to separate plasma and serum from whole blood, and maintenance of a cold chain during transportation and storage.

Both assay types were used in this study (Table 1). The Maxim HIV-1 LAg-Avidity EIA (Maxim Biomedical, Inc., Rockville, Maryland, USA) assays were performed according to the manufacturer’s instructions for use [25]. Wells of a microtiter plate were coated with a limited amount of multi-subtype recombinant HIV-1 antigen to which antibodies bind. Specimens were diluted and incubated for 60 min before a disassociation buffer was added for 15 min to remove weakly-bound antibodies. A goat anti-human, horseradish peroxidase (HRP)-conjugated IgG was then added, which bound to any remaining antibodies. A colour, proportionate to the amount of HRP, was generated by adding a tetramethylbenzidine substrate. Optical densities were measured for each sample and normalised by use of a calibrator specimen. The median was calculated from triplicate testing of the calibrator on each plate. The normalized optical density (ODn) was calculated by dividing the OD for each specimen by the median calibrator OD. Specimens with an ODn value ≤2.0 were retested in triplicate from a fresh dilution, as required by the testing procedures. For these specimens the final ODn value was the median value of the triplicate test results. Anti-HIV serology was performed on specimens showing ODn value < 0.4 to confirm HIV antibody positivity.

### Viral load and ART metabolite testing

Viral load was measured using the laboratory-based Abbott m2000 RealTime system (Abbott Laboratories, Abbott Park, Illinois, USA), Roche Cobas AmpliPrep/Cobas Taqman (Roche Diagnostics International AG, Basel, Switzerland), or similar automated platforms, according to manufacturers’ instructions.

As including information on exposure to ART could improve RITA performance through lowering false-recent misclassification, Nairobi study plasma samples assessed as potentially recent positive were tested for the presence of antiretroviral metabolites at the Division of Clinical Pharmacology at the University of Cape Town. Antiretroviral metabolites, including lopinavir, ritonavir, nevirapine, efavirenz, indinavir, saquinavir, zidovudine, lamivudine, and stavudine, were quantified by a robust simultaneous liquid chromatography/tandem mass spectrometry method [26].

### Evaluation

To monitor the laboratory outputs and ensure testing practices were of good quality, a LAg avidity assay expert (Dr Gary Murphy, expert consultant) reviewed the outputs for a random sample of the plates. This included evaluating the control samples against the permitted criteria, the reproducibility of results, the comparison of initial versus confirmatory results, and the correct entering of results. One of the authors (MdW) met with the study coordinators of the Zimbabwe and Nairobi pilot and observed study enrolment in a clinic as well as the laboratory procedures in Zimbabwe. Towards the end of the study, informal one-to-one discussions were conducted with study coordinators, laboratory leads, and facility-based stakeholders to construct an image of people’s experiences with the different aspects of the project. Discussion topics included experiences with communication, field work, laboratory work, and coordination, as well as recommendations and lessons learned. Together, this provided a picture of how the study was conducted in actuality and how it could be improved in the future. A more detailed description of this study has been provided elsewhere [17].

## Results

Table 2 provides a summary of study recruitment, test results, and return of these results. Across 34 pilot sites, in total 1269 people testing HIV positive completed the RITA, of whom 89 (7.0%) were classified as positive for recent infection. A detailed description of the test results (quantitative focus) have been presented elsewhere [17].

**Table 2:** Recruitment, test results, and return of test results by pilot study

	Siaya County	Nairobi	Zimbabwe^a^
**Participating sites**	14	14	6
**Eligible for HIV test**	2365	50,561	4349
**Tested HIV positive**^b^	445	628	510
**Completed RITA**	426	530	313
**Recent infection positive prior to ART verification**	11 (2.6%)	48 (9.1%)	33 (10.5%)
**Number of recent infections after ART verification**^c^	10 (2.3%)	46 (8.7%)	33 (10.5%)
**Completed RITA results returned to client**	NA		
**Recent infection**		29 (60.4%)	9 (27.3%)
**Long-standing infection**		373 (77.1%)	24 (8.6%)

*NA* not applicable

^a^Results from venous blood tests

^b^Includes people testing HIV positive for the first time in Nairobi and Zimbabwe

^c^ART status was externally verified in Siaya County and Nairobi but not Zimbabwe

### Sample types and transportation

Clear differences were observed in the collection of venous blood and DBS. In our pilot study in Zimbabwe, where both sample types were collected from participants, acceptance rates for recency testing were lower when venous blood collection was required for a sample (*n* = 313, 61%) compared to a finger prick for a DBS sample (*n* = 367, 72%) (*p* < 0.001). After sample collection, venous blood samples needed to be rapidly transported to the laboratory so that they could be centrifuged and stored in a freezer below -20 °C to avoid haemolysis. This proved to be challenging given recruitment sites were often hours from the laboratory and required careful planning. The venous blood and DBS samples were collected and transported at the same time.

In Siaya county, sites were often in rural locations, and venous blood samples were transported by motorcycle in a cooler box in a backpack to avoid damage caused by uneven roads. Transport was daily, as samples had to get to the laboratory within 5 h of collection, and took 1 to 2 h. Measures to reduce transportation time, such as having drivers stationed at a central location and available to all sites, were shown to work as none of the collected samples were rejected by the laboratory for poor quality.

In Nairobi, where sites were closer in proximity, a driver collected samples from all sites for delivery to the laboratory in the morning. If any additional samples were taken later during the day, a courier picked them up from the facilities.

### Laboratory performance

We trained staff to perform the LAg avidity assays correctly. Despite this training, and despite most laboratory staff being familiar with similar assays, laboratory staff in all three settings stated that performing the LAg avidity assay correctly was difficult and required a lot of precision. In one of the pilot studies, we repeatedly encountered invalid results from the LAg avidity assay and had to temporarily pause recruitment during month four of the study. To keep delays to a minimum, we transferred testing to a back-up laboratory during the temporary pause. By comparing operational procedures between the original and back-up laboratories, we were able to identify causes for the failed runs. The main reason for invalid results was found to be incorrect calibration and maintenance of the microplate washer. The washer was replaced as a result of this investigation and testing could be resumed. We learned that ensuring that these items of equipment are available, well maintained, properly calibrated and used correctly are critical steps before testing starts.

In recognition that a number of challenges across the pilots were arising in relation to collecting, testing, and returning samples, we introduced a regular call that brought together the three in-country pilot teams, as well as the study team based in London. Prior to this, we were running the pilots as linked but independent studies. The laboratory technicians appreciated having such a network of expertise as they were able to discuss the problems they encountered, share advice, and gain support.

Another lesson we identified retrospectively was the potential utility of participating in an external quality assurance scheme. A scheme for the performance of Maxim and Sedia LAg assays is run by Duke University’s External Quality Assurance Program Oversight Laboratory (EQAPOL) [27]. This scheme distributes specimens of known reactivity to participants who then return the results of their testing to EQAPOL. These results are analysed to determine agreement with the expected results thereby helping identify whether laboratories are performing tests correctly. Signing up to this early on in our study might have prevented the recruitment pause.

In Table 3, we summarise issues that can occur when performing recency testing in laboratories. We present a combination of challenges we encountered in this study and challenges encountered by our team’s LAg avidity assay expert in similar studies.

**Table 3:** Potential issues when implementing a RITA, with recommendations and additional considerations

	Issue	Recommendation	Additional considerations
**Recency assay**	Assay availability may be limited	Discuss with assay manufacturer well in advance of launch of study the number of assays required.	This will require an understanding of the population to be studied and the anticipated confirmation rate.
	Assay kit may not be able to be imported into country	Ensure import permits are in place at an early stage.	Delays to imports may affect performance of assay if it is not stored in appropriate condition when awaiting customs clearance.
	Assay transport within country	Ensure cold chain is maintained for the assay when it is transported to local labs.	Minimising number of laboratories performing the assay may make this easier.
**Sample types**	Specimen transport within country	Specimens should be transported under cold chain. Serum/plasma specimens for LAg testing should be separated locally before transport. DBS specimens should ensure that the specimen is fully dried before transport and maintained with a desiccant.	Depending upon other tests required, different specimens may need to be transported under different conditions.
**Training and performance**	Before testing is undertaken staff should be trained in the performance of the assay	Although a standard EIA this assay has multiple pass/fail criteria and troubleshooting errors can be complex. All users should receive training from an experienced user before undertaking ‘real-world’ testing. CDC offer training panels to help users achieve competency in the assay and help should be sought from CDC.	This training will not only help support the testing procedure but also the analysis tools of the associated software. Individuals should be monitored regularly to confirm compliance with the testing protocol. Testing laboratories must ensure that Standard operating procedures are in place for the assay detailing all steps and conditions undertaken in their lab to allow troubleshooting of issues and enhance data analysis.
	Assay only requires relatively basic laboratory equipment however it is very sensitive to issues such as inadequate washing.	Ensure all required equipment is available and is fully serviced, maintained and calibrated. Equipment to be used should be itemised before testing begins and reviewed by an experienced individual to ensure it is suitable for use.	Service contracts may not be in place for some pieces of equipment so monitoring the performance of equipment is critical to ensure it is performing as expected. When considering equipment even ‘common’ items such as pipettes should be serviced and calibrated before use.
	Additional items such as bloodletting equipment and Dried Blood spot cards are required	These are often outside of the control of the laboratory, but it should be ensured that the equipment used is of the right type, within expiry date and stored appropriately.	Details of all ancillary equipment should be recorded and available for inspection.
	Reagents should be stored as per assays instructions for use (IFU).	Reagents are stored at a variety of temperatures and these must be adhered to. Temperature monitoring should be in place for all freezers and refrigerators.	Ensure that supplies such as water are appropriate for use following assay’s ‘IFU’.
	In use reagents	Wash reagents must be made as per the assays ‘IFU’. Unless the whole kit is being used at one time then unused reagents must be returned to appropriate storage conditions as soon as possible.	
	Testing must be performed in accordance with the assays ‘instruction for use’	LAg assay is a generic term and at least two manufacturers supply a version of the assay. It is critical that the correct method is used for each assay.	As well as two manufacturers there are different versions of the kit dependent on sample type being used plasma/serum or DBS. The appropriate method must be followed for these.
**Quality control**	Temperature control	LAg assays are very prescriptive on incubation times and temperatures. Assays should be performed using incubators with precise and recorded temperatures rather than incubated in the open lab regardless of the current temperature of the lab.	Regular monitoring of equipment temperatures should be recorded and incubators confirmed to have reached temperature before starting assay.
	Internal quality control	Although the assays come with control material, users should follow good laboratory practice and include some specimens of known reactivity in every test to ensure reproducibility over time.	These results should be plotted and analysed to look for any trends in performance.
	Analysis of quality control data	The manufacturer supplied analysis software will provide quality control data on the assay performance. Users should ensure that they enter data correctly and ensure upper reading OD of the spectrophotometer is accurately entered. Users should review data on their own internal quality control independently as this is not captured longitudinally by company software.	
	External Quality Assurance	Each testing laboratory should partake in an external quality assurance programme for the assay. This will help provide confidence in the results issued by each laboratory. The programme for this assay is available via EQAPOL.	
	Assay failures	Laboratories should record and share any failed runs with assay specialists. This will help to identify if there is any systematic error occurring with the assay and enhance the ability to troubleshoot the assay.	Users should ensure that details of all equipment used (Including pipettes etc) is recorded and the items are identifiable by serial number. Batch number of assay should be recorded (this will be performed by the company supplied software if used).
	Assay linked analysis software	Users should ensure they are using the correct analysis software associated with the assays. Each assay has different validation criteria and use of the incorrect manufacturer’s software may lead to errors in assay interpretation and validation.	Users should also be aware to use the correct software for plasma/serum specimens and for DBS specimens as these differ.
	Confirmatory testing	Confirmatory testing must be performed from a freshly diluted specimen. It must not be performed from the dilution prepared for the screening assay.	Reviewing confirmatory test data can help determine how well an assay is being performed as the replicates should be very close. A wide range of values on the confirmatory test may indicate poor pipetting competency of the tester or poor reliability of the pipettes.
	Unusual results	All unusual results should be investigated, and retesting undertaken if warranted. Unusual results may include specimens which offer a low OD (which require retesting to confirm the specimen contains antibodies to HIV-1) or samples where the screening ODn is significantly different from that of the confirmatory test. Investigations should be undertaken to confirm that the correct specimen was retested (or tested initially).	Many assays will not differentiate HIV-1 from HIV-2 so care should be taken where HIV-2 is prevalent.
**Reporting results**	Confounding factors	Users should be aware that a number of factors can affect the performance of the test and these should be considered when analysing data. These include factors such as anti-retroviral treatment of individuals and HIV subtype prevalence.	
	Assay specific performance data	Users should be very clear as to which assay was used and the cut-offs applied to their data as this impacts on the period in which recent HIV infection can be inferred by the assay. This is also important where multiple HIV-1 clades are prevalent.	Results should not be issued without being part of an algorithm to reduce the potential for misclassification.
	Over-interpretation of the data	Users should be aware of the limitations of the assay and not over interpret the data. For example, a very low ODn may indicate a very recent HIV infection or that the individual is not HIV infected. As per the algorithm these specimens should be submitted for HIV diagnostic confirmatory testing.	Application of data trends should take into account potential confounders such as changes in the population, the application of different HIC screening tests over time and the use of different LAg assays in previous surveys.

### Organisation of fieldwork

Recency testing is increasingly implemented as part of existing programmes and/or services [13]. As described in the methods, we integrated recency testing into antenatal care services, a general HIV testing service, and a sex workers programme. Embedding recency testing in existing structures made it possible to get the study started quickly and also helped to calculate the expected sample size given that the number of clients visiting the programme was known from previous experience. In the Nairobi and Zimbabwe pilots, observed recruitment was similar to expected (as calculated in our original protocols based on programme activity) recruitment (600 expected and 628 recruited in Nairobi; 600 expected and 510 recruited in Zimbabwe). In Siaya County, recruitment was lower than anticipated (864 expected and 445 recruited). The differences between our observed and expected recruitment numbers reflect how the pilot studies were reliant upon existing programme activity. For example, in Zimbabwe, a delay in funding for programme activities resulted in our study commencing later than expected.

Embedding our pilots within established programmes also required, at times, client flows to be adapted and additional staff to be accommodated. The study required the inclusion of additional questions to the routine client question list. The inclusion of recency testing-related questions in some settings lengthened the routine consulting period and may have resulted in increased hesitation by clients to participate in the study. To overcome potential client concerns, staff were trained to help people find their way around the facility and to answer questions as they arose. Additional study requirements in some settings resulted in an extra data clerk having to be accommodated, which in turn resulted in having to adapt clinic-space organisation. The impact of new tests and personnel on clinic organisation can negatively impact client flow [28].

Study recruitment was observed to vary across facilities and time. In Siaya County, routine checks were conducted to see whether we were reaching everybody attending the facilities. This was done by comparing the number of participants enrolled in the study to the number of participants recorded in the antenatal care register. If a potential participant were missed, she was reached during the subsequent visit. In Nairobi, reminders for recruitment were implemented into the electronic medical records system to ensure nobody was missed. In hindsight, it would have been useful to have trained study staff to conduct real-time data monitoring to pick up on variations in recruitment and data entry errors quickly. By doing this, low recruitment or acceptance rates could be identified earlier on, and resolved.

### Return of test results

The timely return of test results to facilities and clients proved to be particularly challenging. We had planned in Nairobi and Zimbabwe to schedule the return of the recency test result to clients during their next clinic visits. However, we found that this was not always feasible, and clients often did not return for their results. In Nairobi and Zimbabwe, respectively, 402 (76%) and 33 (11%) of results were finally returned to the client. In Nairobi, only 7 (1.7%) of results were returned within 1 month and 266 (66.2%) of results were returned more than 3 months after recruitment of the participant. In Zimbabwe, return time was highly variable and mostly dependent on when women returned to the clinic. Women frequently did not return to the clinic for results and gave false contact details which hampered follow-up. The late return of a test result may have a negative impact on the value of the result to the client and their utility in informing prevention activities such as partner notification [19].

Delays in laboratory testing occurred due to failed assay runs or increased waiting time for the samples to be tested when recruitment was slow. As recency testing kits are expensive, we aimed to minimise the amount of reagents used. With every assay using 11 control wells, it proved efficient to run plates only when enough samples were available to fill the plate. During periods when recruitment was slow, this resulted in delays. This not only delayed return of test results but also meant that samples were stored for different durations, potentially affecting results if storage conditions were not optimal.

Another significant challenge to the timely return of test results was the need for multiple assays to complete the RITA. Viral load tests could be run in our participating laboratories, but ART metabolite testing required samples to be sent to the University of Cape Town in South Africa. This proved to be difficult for two reasons: obtaining import and export permits within the study period, and a testing backlog at the University of Cape Town. Although we planned to conduct ART metabolite testing across all three pilots, due to these two challenges, it ended up only being carried out for the Nairobi pilot. Having an in-country facility that can conduct ART metabolite testing would have helped overcome these issues, although this might not be worth the costs unless it were part of a holistic infrastructure development. Apart from additional administrative and logistical work, shipment for ART metabolite testing also required high standards of storage. This raises the question of what the optimal balance is between completing the full RITA and quickly returning test results to participants. An important consideration for a RITA result that lacks an ART test component is the information and counselling provided upon the return of recency test result to a client. The interpretation of a test result is complicated regardless of the results because of the long window of recent infection and the risk of false recent results. This becomes even more complicated when exposure to ART in unknown and has to be kept in mind when giving feedback to clients.

## Discussion

We conducted three pilot studies of HIV recency testing in routine service-provision settings in Kenya and Zimbabwe. A total of 1269 people completed the RITA across the three studies, of whom 7% were found to have a recent infection. The main implementation challenges we faced related to sample collection and handling, LAg avidity assay performance, integration into existing services, and returning of test results to clients. In this paper, we provide a detailed analysis of challenges related to laboratory procedures, together with recommendations on how to address, and preferably avoid, these.

The choice to use DBS or venous blood samples affected acceptance rates, transportation, and storage. Although the acceptance rate for venous blood was lower than for DBS in Zimbabwe, challenges relating to the rapid transportation and storage of venous blood samples were overcome through careful planning and coordination. Ensuring the correct performance of the LAg avidity assay and obtaining high quality results were more difficult than expected, underscoring the need for adequate training and competency assessment of staff members performing the assay. When problems with equipment came to light, having a network of laboratories and experts working together was useful to troubleshoot problems, promote collaboration and provide back-up services. Because the pilots were integrated into existing services, the study could start relatively quickly. However, we also came across the downside of being dependent on other programmes and having to adapt routine practices. Returning RITA test results to participants in a timely manner proved to be particularly challenging due to delays in laboratory testing, the need for multiple test results, alignment with clinic visits, and participants not returning for test results. As late return of test results may lessen the value of the results for clients, and for informing activities such as partner notification, future programmes planning to return test results should take account of these issues in their planning stage. Return of the test result had no impact on treatment, as all participants that tested HIV positive were initiated on an ART regimen, regardless of their recency status.

Our study was one of the first to implement recency testing in routine settings in sub-Saharan Africa. As recency testing is currently being scaled up in PEPFAR-supported countries to include all people with newly diagnosed HIV infection [13], our real-world experience and lessons learned can provide helpful guidance to the implementation of HIV recency testing.

## Conclusions

Our analysis showed that recency testing can be successfully implemented in routine settings. However, a number of challenges can be expected: challenges that can be overcome through careful preparation, continuous monitoring of test results to ensure high quality, good communication, intensive coordination with routine service providers, and quick turn-around of laboratory tests.

## Data Availability

The datasets generated and/or analysed during the current study are not publicly available due their sensitive nature but are available from the corresponding author on reasonable request.

## Abbreviations

ART: Antiretroviral therapy
CDC: Centers for Disease Control and Prevention
CeSHHAR: Centre for Sexual Health and HIV/AIDS Research
DBS: Dried blood spot
EDARP: Eastern Deanery AIDS Relief Programme
EIA: Enzyme-linked immunoassay
EQAPOL: External Quality Assurance Program Oversight Laboratory
HDSS: Health and Demographic Surveillance Site
HRP: Horseradish peroxidase
IFU: Instructions for use
KEMRI: Kenya Medical Research Institute
LAg: Limiting antigen
ODn: Normalized optical density
PEPFAR: U.S. President’s Emergency Plan for AIDS Relief
RITA: Recent infection testing algorithm
WHO: World Health Organisation
